# Investigating the role of bentonite clay with different soil amendments to minimize the bioaccumulation of heavy metals in *Solanum melongena* L. under the irrigation of tannery wastewater

**DOI:** 10.3389/fpls.2022.958978

**Published:** 2022-09-29

**Authors:** Waqas ud Din Khan, Xiangying Wei, Hafiz Haider Ali, Faisal Zulfiqar, Jianjun Chen, Rashid Iqbal, Muhammad Saqlain Zaheer, Basharat Ali, Sana Ghafoor, Umm e. Rabiya, Muhammad Waqas, Rabia Ghaffar, Walid Soufan, Ayman El Sabagh

**Affiliations:** ^1^Sustainable Development Study Centre, Government College University, Lahore, Pakistan; ^2^Tasmanian Institute of Agriculture, University of Tasmania, Hobart, TAS, Australia; ^3^College of Geography and Oceanography, Institute of Oceanography, Minjiang University, Fuzhou, China; ^4^Department of Horticultural Sciences, Faculty of Agriculture and Environment, The Islamia University of Bahawalpur, Bahawalpur, Pakistan; ^5^Department of Environmental Horticulture and Mid-Florida Research and Education Center, Institute of Food and Agricultural Sciences, University of Florida, Apopka, FL, United States; ^6^Department of Agronomy, Faculty of Agriculture and Environment, The Islamia University of Bahawalpur, Bahawalpur, Pakistan; ^7^Department of Agricultural Engineering, Khwaja Fareed University of Engineering and Information Technology, Rahim Yar Khan, Pakistan; ^8^Department of Physics, Government College University, Lahore, Pakistan; ^9^Division of Science and Technology, Department of Botany, University of Education, Lahore, Pakistan; ^10^Plant Production Department, College of Food and Agriculture Sciences, King Saud University, Riyadh, Saudi Arabia; ^11^Department of Agronomy, Faculty of Agriculture, Kafrelsheikh University, Kafr El-Shaikh, Egypt

**Keywords:** bentonite, growth parameters, soil chromium, *Solanum melongena*, wastewater

## Abstract

Wastewater from tanneries is a major source of heavy metals in soil and plants when used for crop irrigation. The unavoidable toxicological effects of this contamination, however, can be minimized through two independent steps discussed in the present study. In the first step, a batch sorption experiment was conducted in which Cr was adsorbed through bentonite clay. For this purpose, DTPA extraction method was used to analyze Cr concentration in the soil after regular time intervals (0.5, 1, 2, 6, 8, 9, 10.5, 11.5, and 20.3 h) which reduced Cr concentration from 38.542 mgL^–1^ for 30 min to 5.6597 mgL^–1^ for 20.3 h, respectively, by applying 1% bentonite. An increase in the contact time efficiently allowed soil adsorbent to adsorb maximum Cr from soil samples. In the second step, a pot experiment was conducted with 10 different treatments to improve the physiological and biochemical parameters of the *Solanum melongena* L. irrigated under tanneries’ wastewater stress. There were four replicates, and the crop was harvested after 30 days of germination. It was seen that the application of wastewater significantly (*P <* 0.01) reduced growth of *Solanum melongena* L. by reducing root (77%) and shoot (63%) fresh weight when compared with CFOP (Ce-doped Fe_2_O_3_ nanoparticles); chlorophyll a and b (fourfolds) were improved under CFOP application relative to control (CN). However, the deleterious effects of Cr (86%) and Pb (90%) were significantly decreased in shoot through CFOP application relative to CN. Moreover, oxidative damage induced by the tannery’s wastewater stress (*P <* 0.01) was tolerated by applying different soil amendments. However, results were well pronounced with the application of CFOP which competitively decreased the concentrations of MDA (95%), H_2_O_2_ (89%), and CMP (85%) by efficiently triggering the activities of antioxidant defense mechanisms such as APX (threefold), CAT (twofold), and phenolics (75%) in stem relative to CN. Consequently, all the applied amendments (BN, BT, FOP, and CFOP) have shown the ability to efficiently tolerate the tannery’s wastewater stress; results were more pronounced with the addition of CFOP and FOP+BT by improving physiological and biochemical parameters of *Solanum melongena* L. in an eco-friendly way.

## Introduction

The industrial contamination of air, soil, and water by potentially toxic elements (PTEs) poses a serious threat to human life and the environment (Luo et al., 2020; Awad et al., 2021). In general, it is noticed that PTEs affect the soil’s biological and physiochemical properties, causing issues such as low fertility, low soil organic matter, extreme pH, low nitrogen and phosphorous availability, high electrical conductivity, and micronutrient imbalance (Zaheer et al., 2021, 2022). Similarly, plant growth and its physiology are severely affected by PTEs pollution, a serious global environmental concern (Xie et al., 2016). The main negative impacts on plant growth are nutrient unavailability, stunted root growth, reactive oxygen species (ROS) production, and lower food availability due to lack of photosynthesis (Xie et al., 2016; Nazli et al., 2020). In plants, PTEs toxicity shows a complex series of metal interactions with signal transduction pathways and genetic processes at the cellular level which causes genetic mutation and programmed cell death (Kumari et al., 2016). Among different PTEs, Cr-toxicity in plant tissues may provoke significant changes in biochemical and morpho-physiological processes; this then disturbs their essential metabolic processes (Kamran et al., 2017; Sharma et al., 2020). Cr-toxicity minimizes plant growth by affecting the ultrastructural modifications of chloroplast and cell membrane, reducing pigment content, affecting transpiration, altering different enzymatic activities, and damaging root cells (Farooq et al., 2016; Gill et al., 2016; Anjum et al., 2017; Sharma et al., 2020; Manzoor et al., 2022). Different studies revealed that Cr and Pb stress are also responsible for the production of ROS and MDA contents in plants which cause the damaging effects on their internal structure (Ali et al., 2014a,b; Gill et al., 2015; Aslam et al., 2021).

These threats might be reduced by applying different amendments like bentonite, iron nanoparticles, and bacterial species to boost the soil quality, which ultimately results in better growth of plants. Cost-effective *in situ* soil remediation techniques are widely used to immobilize PTEs and reduce their bioavailability by precipitation, sorption, and complexation mechanisms (Shrivastava et al., 2018). Clay minerals and inorganic composite materials have been proven as efficient adsorbents of PTEs from wastewater (Wahba et al., 2017). Moreover, bentonite clay is a cost-effective way to remove PTEs in soil due to its permanent charges at its surfaces, isomorphic substitution, and environmental compatibility and ready availability (Xie et al., 2018; Mi et al., 2020). Similarly, literature revealed that the addition of 0.5 g bentonite clay to 10 mgL^–1^ PTEs solution efficiently decreased the concentrations of Cu (87%) and Pb (89%) ions (Hussain and Ali, 2021). Similarly, the effect of bentonite on the adsorption of PTEs from wastewater is also well known (Li et al., 2016). Microbes immobilize the metals using different *ex-situ* or *in-situ* mechanisms such as biosorption, metabolism, precipitation, and bioaccumulation (Kapahi and Sachdeva, 2019). Their interaction with metals depends on different soil physiochemical factors such as pH, temperature, moisture content, available ions, and other competitor organisms/species (Kapahi and Sachdeva, 2019). However, *Azotobacter nigricans* strain NEWG-1 is widely used because it is an efficient bio-sorbent for metal removal, cost-effective, reliable, and eco-friendly. *Azotobacter nigricans* strain NEWG-1 (bacterial species) has previously been reported to remove maximum Cu^2+^ as 80% under 200 mgL^–1^ CuSO_4_ solution conditions with a 4-day incubation period of pH 8 (Ghoniem et al., 2020).

In recent years, nanotechnology has emerged as a valuable tool for environmentalists for removing pollutants from the air, water, and soil; indeed, this field has gained more importance than the other traditional methods (Yu et al., 2021). There are two main advantages of using nanotechnology for soil reclamation: higher reactivity due to smaller particle size and larger surface area and easier delivery of small-sized particles into soil pores (Tafazoli et al., 2017). However, researchers have referred to iron-based nanoparticles as good adsorbents of PTEs from soil (Tafazoli et al., 2017; Zand and Tabrizi, 2021). They convert PTEs to less toxic forms, immobilize them, or reduce their availability to plants (Rui et al., 2016). Moreover, CeO_2_ nanoparticles may also enhance root growth and activate the antioxidant enzymes as well as help in the prevention of ion leakage and membrane peroxidation (Cao et al., 2018). Although literature has explored how to remediate heavy metal toxicity with the applications of nanoparticles, its interaction with bacterial species and bentonite is still lacking. In the present study, two independent experiments were carried out. Firstly, we aimed to optimize the rate of bentonite against concentrated wastewater slurry at different time intervals. Then, the selected rate of bentonite with *Azotobacter nigricans* was applied to the soil with brinjal (*Solanum melongena* L.) grown in it and a foliar application of iron oxide nanoparticles given at a later growth stage. More specifically, we aimed to investigate the potential of bentonite clay against industrial wastewater through the application adsorption isotherms and kinetics models and to perform a comparative analysis by assessing the microbial activity of *Azotobacter nigricans* with the inorganic amendments to combat PTEs in improving *Solanum melongena* L. growth, physiology, and biochemical properties. It was hypothesized that the proposed research study might significantly enhance soil fertility and overall yield of plant without compromising the environment.

## Materials and methods

### Wastewater collection and characterization

Wastewater was collected from Siddiq Leather Works Pvt. Ltd., located at 13-km Sheikhupura Road Lahore, Pakistan, at 31°37′48.02 ″E and 74°13′01.25 ″N; characteristics of this water is given Table 1. Before wastewater analysis, the water was passed through a filtration step to remove all solid content (suspended solid, filterable solids, and total dissolved solids). Then, different concentrations of PTEs and heavy metals (Supplementary Table 1) were measured using Atomic Absorption Spectrophotometer (AAS) (Thermo Scientific ICE-3000 series) which used acetylene gas for analysis.

**TABLE 1:** Characterization of tannery wastewater used in the experiment.

Parameters	Tanneries wastewater	Parameters	Tanneries wastewater
DO (mg/L)	2.65	Pb (mg/L)	0.1815
TDS (mg/L)	21,100	Cu (mg/L)	0.4211
TSS (mg/L)	1,243	Fe (mg/L)	14.654
BOD (mg/L)	4,404	Na (mg/L)	12,002
EC (μS/cm)	43,200	Cr (mg/L)	11.122
COD (mg/L)	12,670	Cd (mg/L)	0.0031
pH	8.4	Zn (mg/L)	1.4753
Cl- (mg/L)	13.5	Ni (mg/L)	0.1525

### Batch experiment design (Study I)

A batch experiment was carried out in the Sustainable Agriculture laboratory of the Sustainable Development and Study Center (SDSC) to determine the adsorption of PTEs onto the surface of adsorbent (bentonite) in metal-loaded contaminated soil. The purpose of this experiment was to assess the effects of six varying bentonite rates (1, 2, 3, 4, 5, and 6%) at nine different contact hours (0.5, 1, 2, 6, 8, 9, 10.5, 11.5, and 20.3 h) of the metal removal efficiency. For the experimental set-up, plastic cups were rinsed with distilled water to remove any contamination, and were then air-dried. Each cup was filled with 100 g of sample soil. After filling the cups, each bentonite rate was applied in all the cups and thoroughly mixed with soil; then, the soil was artificially contaminated with 100% concentrated tannery wastewater. All the experimental cups were kept under controlled conditions of 23°C and humidity of 36%.

### Soil harvesting and quantification of Cr

After a fixed contact/interval time for each replicate, 10 g of soil was taken out of each cup and then measured through weighing balance. It was mixed with 20 mL of DTPA (Diethylene Triamine Penta Acetic Acid) extraction solution (DTPA Protocol from ICARDA manual) in conical flasks. After shaking for 2 h, the solution was filtered, and the concentration of Cr in samples was detected using AAS (Table 2).

**TABLE 2:** Concentrations of total Cr for the treatments (T1 = 1% bentonite, T2 = 2% bentonite, T3 = 3% bentonite, T4 = 4% bentonite, T5 = 5% bentonite, T6 = 6% bentonite) in batch adsorption experiment.

Time (h)	Time (min)	T_1_ (mg L^–1^)	T_2_ (mg L^–1^)	T_3_ (mg L^–1^)	T_4_ (mg L^–1^)	T_5_ (mg L^–1^)	T_6_ (mg L^–1^)
0.5	30	38.54	29.75	27.30	29.12	29.81	29.17
1	60	27.00	20.92	24.40	23.81	21.07	24.47
2	120	15.04	15.45	12.09	14.53	17.57	16.27
6	340	12.71	11.83	11.01	13.27	15.86	13.88
8	480	11.39	9.32	10.78	12.87	12.78	11.20
9	540	8.83	8.75	9.57	6.75	10.10	10.71
10.5	630	7.11	6.08	6.14	5.88	7.07	9.77
11.5	690	7.58	5.11	5.94	5.19	4.91	7.29
20.30	1,218	5.65	4.12	5.31	4.96	3.66	6.10

### Langmuir and Freundlich adsorption isotherms

Langmuir and Freundlich’s adsorption isotherms were applied with pseudo-first-order and pseudo-second-order kinetics to determine sorption isotherms for adsorbents (Manzoor et al., 2013). Expression for log form of Freundlich and Linear form of Langmuir is given in equations 1 and 2:


(1) $${\text{C}}_{\text{e}}/{\text{q}}_{\text{e}}\,=\,1/{\text{q}}_{\max}{\text{K}}_{\text{L}}\,+\,{\text{C}}_{\text{e}}/{\text{q}}_{\max}$$

(2) $${\text{Logq}}_{\text{e}}\,=\,1/{\text{n(LogC}}_{\text{e}})\,+\,{\text{LogK}}_{\text{F}}$$

q_e_ is the amount of adsorbed metal ions (mg kg^–1^), K_L_ is the Langmuir equation constant, and K_F_ and 1/n are Freundlich equation constants.

### Adsorption kinetics models

Adsorption kinetics, i.e., pseudo 1st order and pseudo 2nd order, was applied to the experimental data for the best-fitted adsorption model (Angkawijaya et al., 2020).

The expression for pseudo-first-order can be written as shown in equation 3


(3) $${\text{Log(q}}_{\text{e}}\,-\,{\text{q}}_{\text{t}})\,=\,\log{\text{q}}_{\text{e}}\,-\,\,-({\text{k}}_{1}/2.303)\text{t}$$

where K_1_ is the pseudo-first-order rate constant (min^–1^), q_e_ is the adsorption capacity (mg g^–1^), and q_t_ is the adsorbed metal ion (mg g^–1^) at time t (interval time). The expression for pseudo-second-order is given in equation 4


(4) $${\text{t/q}}_{\text{t}}\,=\,1/{\text{K}}_{2\text{qe}}{}^{2}\,+\,{\text{t/q}}_{\text{e}}$$

where k_2_ is pseudo second-order rate constant (mg g^–1^ min^–1^).

### Preparation of bacterial species, Fe_2_O_3_, and Ce-doped Fe_2_O_3_ nanoparticles

Pure culture of bacterial species *Azotobacter nigricans* sp. (FCBP-PB-0422) was acquired from the First Fungal Culture Bank of Pakistan (FCBP) at the Faculty of Agricultural Sciences, University of the Punjab, Lahore. A method suggested by Loutfi et al. (2020) was followed to culture bacterial species in broth for 48 h (to get more biomass of bacterial culture). More than 99% pure iron (III) nitrate non-ahydrate [Fe(NO_3_)_3⋅_9H_2_O), cerium (III) nitrate (Ce(NO_3_)_3⋅_6H_2_O] were purchased from Sigma-Aldrich, and oxalic acid (C_2_H_2_O_4⋅_2H_2_O) was procured from Duksan. To prepare Fe_2_O_3_ and Ce-doped Fe_2_O_3_ nanoparticles, the easy and economical autocombustion method (Aruna and Mukasyan, 2018) was used. The Fe_2_O_3_ nanoparticles were synthesized by dissolving an 89.7911 g of Fe(NO_3_)_3⋅_9H_2_O in 500 mL of deionized water (DIW). After heating at 120°C for 30 min, 90 g of oxalic acid was added. On heating further for 3 h, a gel-like material was formed that changed into dry ash. The initially prepared material was first calcinated at 250°C for 2 h, then sintered at 800°C for 2 h, and finally pestled. A slightly darker reddish-brown powder of Fe_2_O_3_ nanoparticles (FOP) was collected.

The same autocombustion route was followed to prepare 1% Ce-doped Fe_2_O_3_ nanopowder. Two solutions, A and B, were prepared by dissolving 88.89 g of Fe(NO_3_)_3⋅_9H_2_O in 500 mL and 0.964 g of Ce(NO_3_)_3⋅_6H_2_O in 50 mL of DW, respectively. After heating separately at 120°C for 30 min, both solutions were mixed and heated further for 30 min at 120°C. At this stage, 90 g of oxalic acid was added to the mixture solution and heated at 120°C. On heating further for 1 h, the mixture solution first emerged as a gel and then became ash-like. The obtained material was first calcinated at 250°C for 2 h and then sintered at 800°C for 2 h. Light reddish-brown Ce-doped Fe_2_O_3_ nanoparticles (CFOP) were collected in powder form.

### Experimental design for greenhouse trial (Study II)

The pot experiment was carried out in the greenhouse of the Botanical Garden of Government College University Lahore under partially controlled conditions. *Solanum melongena* L. was taken for the experimental study, considering the morphology and economic importance. For this experiment, the sandy loam soil was used with the following properties: [Soil texture (Clay loam), Sand (37%), Silt (33%), Clay (30%), Organic matter (0.81 %), pH (7.47), EC (1.35 dSm^–1^), CEC (5.86 cmol Kg^–1^), DTPA-Cr (0.2018 mg Kg^–1^), and DTPA-Pb (0.6873 mg Kg^–1^) (Robertson et al., 1999)]. The 4 kg of soil was filled out in each pot. The treatments used in the pot experiment were: T_1_ = Control (CN); T_2_ = Bentonite (BN); T_3_ = Iron Oxide nanoparticles (FOP); T_4_ = Cerium doped iron oxide nanoparticles (CFOP); T_5_ = *Azotobacter nigricans* (BT); T_6_ = BN + FOP; T_7_ = BN + CFOP; T_8_ = BN + BT; T_9_ = FOP + BT; and T_10_ = CFOP + BT. Each treatment was replicated four times and distilled water was frequently applied to maintain soil moisture content. Bentonite was applied at 10 g per kg of soil, iron oxide nanoparticles, and cerium-doped iron oxide nanoparticles was foliar applied at 20 mg L^–1^. *Azotobacter nigricans* strain was inoculated with the seeds according to the treatments. After thorough mixing of amendments in soil, PTEs stress in the form of wastewater (1,200 mL per pot with 10% dilution of wastewater slurry) was added into each pot. After 2 days, the seedlings were planted in the soil. After 6 days of seed germination, diammonium phosphate (DAP) and urea fertilizers were applied at 200 and 150 kg acre^–1^ in every pot to maintain plant nutrients’ balance. After 30 days of germination, plants were carefully harvested and the roots and shoots of each replicate were washed with distilled water to remove the dust particles. Further analysis was carried out in the laboratory using labeled zipper bags.

### Determination of potentially toxic elements (Cr and Pb) in plants

The concentrations of Cr and Pb in all plant parts were determined with the acid digestion method. For this purpose, washed plants were sun-dried for 48 h and oven-dried at 75°C to remove moisture content till a constant weight was achieved. For dry weight analysis to assess the nutrients of dry plants, 0.5 g root and shoot were digested using a di-acid mixture (HNO_3_: HCLO_4_ = 2:1) at 180°C till white fumes appeared (Dad et al., 2021). The samples were then filtered, and the filtrate volume was raised to 50 mL. Multi-sequential Atomic Absorption Spectrophotometer was used to quantify Cr and Pb uptake by plants after the application of soil amendments.

### Determination of chlorophyll concentration in brinjal plant

The chlorophyll content of plants was determined following the method of Strain and Svec (1966). For this purpose, leaf extract was prepared using 0.1 g washed leaf ground with 80% acetone solution. After grinding the leaves, 5 mL of acetone was added to each leaf extract and centrifuged (HERMILE Z167M) at 4,000 rpm for 5 min. The supernatant was then used to determine the absorbance at 663 nm and 645 nm using a spectrophotometer (Shimadzu UV-1201, Kyoto, Japan). The concentration of chlorophyll a and b were calculated using the equation by Strain and Svec (1966).


(5) Chl⁢ a⁢ (mg/mL)⁢ = (11.64*A663)−(02.16*A645)

(6) Chl⁢ b⁢ (mg/mL)⁢ = (−3.94*A663)+(20.97*A645)

A663 = absorbance at 663 nm wavelength and A645 = absorbance at 645 nm wavelength (Strain and Svec, 1966).

### Malondialdehyde and hydrogen peroxide

Malondialdehyde (MDA) were measured by taking 0.5 g of fresh root, stem, and leaf and grinding this with 5 mL of trichloroacetic acid (TCA) solution. The prepared extract was centrifuged at 10,000 rpm for 10 min. After centrifugation, 2.5 mL of supernatant was mixed with 1 mL of 0.5% thiobarbituric acid (TBA) in 20% TCA solution. The mixture was then placed in a dry oven (Memmert ULM-400) at 95°C for 30 min. It was allowed to cool down and absorbance was checked at 532 and 600 nm using a spectrophotometer (Jambunathan, 2010).

For measurement of hydrogen peroxide (H_2_O_2_) concentration in a sample plant, 0.5 mL of supernatant of roots, stem, and leaf extract was added to 5 mL of potassium phosphate (K-P) buffer. The mixture was centrifuged at 10,000 rpm for 10 min. After centrifugation, the supernatant was mixed with 5 mL of 0.1% TCA (w/v) and 1 mL of potassium iodide (KI) buffer. The absorbance of the mixture was measured at 390 nm using a spectrophotometer (Velikova et al., 2000).

### Cell membrane permeability

Cell membrane permeability (CMP) was analyzed by taking 0.5 g tissues of leaves, stem, and roots in 10 mL of distilled water and allowed to shake for 24 h. After continuous shaking for 24 h, electrical conductivity (EC1) was measured using an EC meter (Extech EC300). The heterogeneous mixture was then autoclaved for 20 min at 121°C (Jambunathan, 2010). After autoclaving, EC2 was measured again, and the CMP was calculated using the following formula:


(7) Electrolyte⁢Leakage=(EC1/EC2)*100

### Determination of antioxidant enzymes (APX and CAT)

Sample extract for the determination of antioxidant enzymes (APX and CAT) was prepared by taking 0.2 g of the fresh root, stem, and leaf, which was then homogenized in 1.2 mL of 0.2 M K-P buffer with pH 7.8, and 20 min were given for the centrifugation at 15,000 rpm and 4°C temperature. Then, the supernatant was separated, and the pellet was collected and re-suspended in the same buffer with 0.8 mL volume. For 15 min, the suspension was again centrifuged at 15000 rpm. Finally, different activities of antioxidant enzymes were measured by combining both supernatants and storing them at freezing temperatures (Cakmak and Marschner, 1992).

To determine APX of root, stem, and leaf, a 40 μL extract was mixed with 1,320 μL of 50 mM KH_2_PO_4_ buffer solution, 1,320 μL of 0.5 mM ascorbate, and 0.5 mM H_2_O_2_. Absorbance was noted using a spectrophotometer at 290 nm (Nakano and Asada, 1981). For CAT analysis, each plant’s root, stem, and leaf extract was diluted 200 times with 50 mM KH_2_PO_4_. Then, 1 mL of supernatant was separated and mixed with 2 mL of H_2_O_2_ (Cakmak and Marschner, 1992). Absorbance was determined at 290 nm using a spectrophotometer (Shimadzu UV-1201, Kyoto, Japan).

### Total phenolics and protein

Total phenolics content in root, stem, and leaf was determined using protocols as described previously (Singleton et al., 1999; Sultana et al., 2009). For this purpose, 0.1 g of root, stem, and leaf samples were ground using 4 mL of acetone. A 60 μL of sample extract was mixed with 4,740 μL of distilled water, 300 μL of Folin-Ciocalteu reagent, and 900 μL of Na_2_CO_3_ (1N). The test tubes were then placed in an oven at 55°C. Absorbance was determined at 760 nm using a spectrophotometer (Shimadzu UV-1201, Kyoto, Japan). The phenolics content was calculated in μg g^–1^. Protein concentrations of root, stem, and leaf were determined using the Bradford method (1976). A 200 μL extract was mixed with 1,800 μL of distilled water and 2 mL of Bradford reagent. Test tubes were incubated for 15 min at 80°C. The absorbance was determined at 595 nm using spectrophotometer (Shimadzu UV-1201, Kyoto, Japan).

### Statistical analysis

Both the experiments were conducted in a completely randomized design and data was analyzed by different statistical analytics. For this purpose, Microsoft Excel 2016 (Microsoft Cooperation, USA) was used to analyze the dataset for different statistical operations such as average and standard deviation. One-way ANOVA and the least significant difference (LSD) were performed on the dataset using Statistix (JMP^®^ Analytical Software 8.1, Tallahassee, USA) to interpret the results (Ramzani et al., 2017a,b). Finally, Sigmaplot (14.0) was used for the graphical representation of the results. The results shown in this manuscript are the average values of four (*n* = 4) replicates with standard errors (SE).

## Results

### Adsorption isotherm models

The results indicated that the Langmuir isotherm model was best fitted for this study’s experimental data, which assumes that Cr ions formed a monolayer on the surface of the adsorbent (Table 3). Q_max_ was highest in T_2_, while all other treatments represented an exponential decrease in Q_max_. To interpret the results and reliability of best-fitted reaction kinetic models for experimental data, the basic criteria used is comparing q_e.cal_ with q_e.exp_ and *R*^2^ value. In this methodology q_e.cal_ was closely related to q_e.exp_ for both reaction kinetic models (PFO and PSO). However, coefficient of determination values differs significantly in both models (Table 2). Below coefficient of determination (*R*^2^) has higher values for the Pseudo-2nd order than the Pseudo 1st order. This indicates that Pseudo 2nd order is more reliable in describing the experimental data of the study. In most research studies in the past, the 2nd order kinetic model has been more reliable and accurate than Pseudo 1st order in providing correlation with experiment (Simonin and Bouté, 2016).

**TABLE 3:** Comparison of Langmuir and Freundlich isotherm models for different concentrations of adsorbents and pseudo 1st and 2nd order in terms of their *R*^2^-values for the treatments (T1 = 1% bentonite, T2 = 2% bentonite, T3 = 3% bentonite, T4 = 4% bentonite, T5 = 5% bentonite, T6 = 6% bentonite).

Treatments	Langmuir		Freundlich		1st order			2nd order		
	Qmax (mg/kg)	*R* ^2^	Q(max) (mg/kg)	*R* ^2^	K_1_	*R* ^2^	q_e_ (mg/g)	K_2_	*R* ^2^	q_e_ (mg/g)
T1	12.99	1	3.07	0.93	0.01	0.80	17.40	0.78	1	20.1
T2	52.91	1	3.09	0.91	0.01	0.88	1.34	0.77	1	94.14
T3	36.99	1	3.05	0.94	0.01	0.78	0.97	0.78	1	54.27
T4	14.99	1	3.05	0.93	0.01	0.79	0.74	0.57	1	43.74
T5	16.96	1	3.05	0.98	0.01	0.90	7.14	0.76	1	27.17
T6	10.93	1	3.06	0.94	0.01	0.88	0.14	0.64	1	23.43

### Structural analysis of Fe_2_O_3_ and Ce-doped Fe_2_O_3_ nanoparticles

**FIGURE 1:**
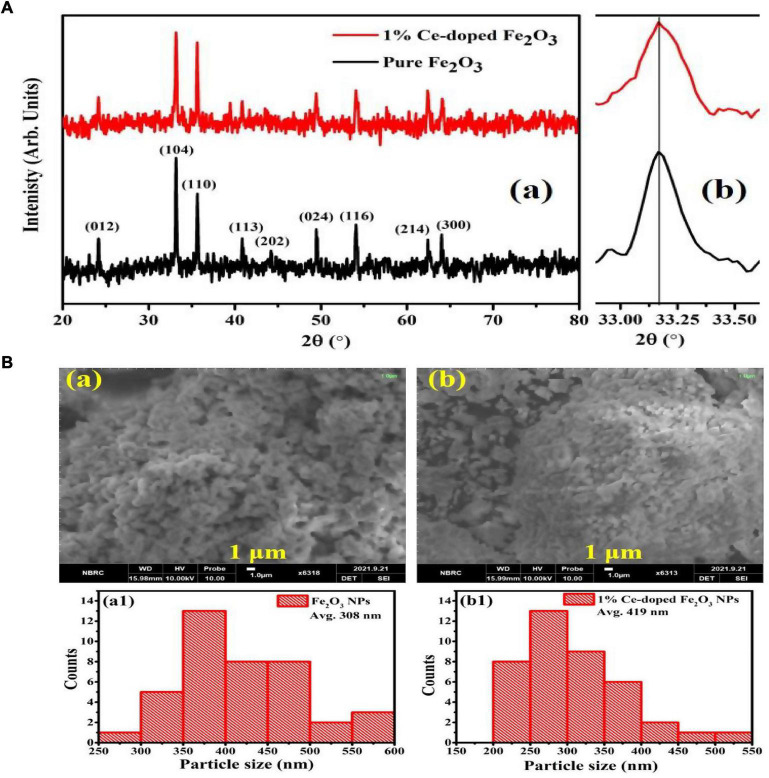
**(A)** X-ray diffraction profiles of undoped Fe_2_O_3_ and Ce-doped Fe_2_O_3_ nanoparticles synthesized by auto combustion method using oxalic acid (a). Slight shifting of Fe_2_O_3_ (104) peak, to lower 2θ angles, is due to Ce-atoms doping (b). **(B)** SEM images of pure Fe_2_O_3_ (a) and 1% Ce-doped Fe_2_O_3_ NPs (b). Particle size distributions of pure Fe_2_O_3_ (a1) and 1% Ce-doped Fe_2_O_3_ NPs (b1).

X-ray diffraction graphs of Fe_2_O_3_ (FOP) and Ce-doped Fe_2_O_3_ (CFOP) nanoparticles are shown in Figure 1. The crystalline phase present in FOP and CFOP confirms the successful synthesis. Due to the low Ce amount, the shifting of diffraction peaks is not prominent, as shown in Figure 1Ab. In the diffraction patterns of FOP and CFOP, the peaks at 21.18°, 35.58°, 40.8°, 49.5°, 54.03°, 62.5°, and 63.9° correspond to (102), (104), (110), (113), (024), (116), (214), and (300) crystallographic planes, respectively, and indexed with JCPDS card no. 39-1346 (Wahab et al., 2018). The sharpness of the Bragg peak corresponds to the excellent crystallinity achieved in the synthesis of both materials. Moreover, the absence of any secondary phase confirms the proper doping of the Ce inside the Fe_2_O_3_ lattice. The Ce-atoms in the Fe_2_O_3_ structure leads to the lattice distortions responsible for the stability of Fe-O bonds in the doped materials (Ning et al., 2021). Based on the Debye-Scherrer relation $\text{D}\,\frac{\text{k}\,\lambda }{\beta \,\cos\theta }$, the average crystallite size obtained for the undoped Fe_2_O_3_ and Ce-doped Fe_2_O_3_ is 37.29 and 39.61 nm, respectively.

### Morphology of nanoparticles

Scanning electron micrographs and particle size distributions of the prepared FOP and CFOP nanoparticles are shown in Figures 1Ba–d, 2. Agglomeration of nanoparticles can be seen in both FOP and CFOP, with an average particle size of 308 and 419 nm, respectively (Ning et al., 2019).

**FIGURE 2:**
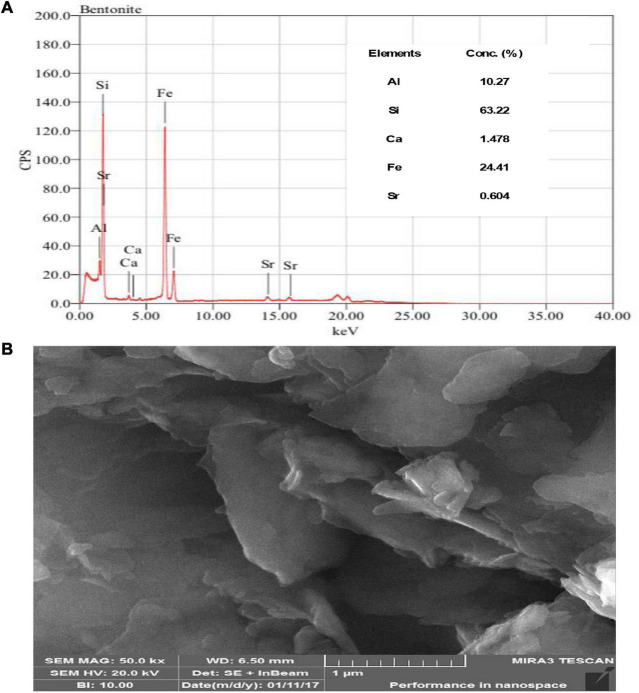
**(A)** X-Ray fluorescence image of bentonite clay presented the elemental concentration while **(B)** SEM images of bentonite clay presented the structural and physical appearance at 1 um.

### Scenario of growth parameters

Wastewater application significantly (*P <* 0.01) decreased the growth and development of *Solanum melongena* L. (Table 4). However, the application of BN, BT, FOP, and CFOP both alone and in combinations with each other significantly (*P <* 0.01) influenced the fresh weight, dry weight, and chlorophyll content of *Solanum melongena* L. (Tables 4, 5). The fresh weight of root competently increased (threefold) under the treatment of CFOP when compared with CN (Table 4). Interestingly, shoot fresh weight was significantly (*P* < 0.01) increased (onefold) through the application of FOP + BT as compared with CN, but the maximum fresh weight was also observed under CFOP (twofold) as compared with CN in the shoot of *Solanum melongena* L. (Table 4). Similarly, total biomass was significantly (*P* < 0.01) influenced by the application of different soil amendments under wastewater stress (Table 4). The applications of BN and FOP + BT efficiently increased (75%) the root dry weight when compared with CN; however, a maximum increase (onefold) in shoot dry weight was observed under CFOP as compared with CN in *Solanum melongena* L. (Table 4). Moreover, the chlorophyll contents of the *Solanum melongena* L. were significantly (*P <* 0.01) decreased by the application of tannery wastewater (Table 5). However, the application of CFOP and FOP + BT efficiently increased (fourfold) the content of chlorophyll a in *Solanum melongena* L. when compared with CN (Table 5). Interestingly, the maximum increase (fourfold) in chlorophyll b of *Solanum melongena* L. was noticed through CFOP application when applied alone compared with CN under wastewater stress (Table 5).

**TABLE 4:** Dry and fresh weight (g) and length (cm) of root and shoot of brinjal plant *(Solanum melongena)* as affected by various treatments: Control (CN); bentonite (BN); Iron Oxide nanoparticles (FOP); Cerium-doped iron oxide nanoparticles (CFOP); *Azotobacter nigricans* sp. (BT); BN + FOP; BN + CFOP; BN + BT; FOP + BT; T10 = CFOP + BT.

Treatments	Fresh weight (g)		Dry weight (g)		Length (cm)	
	Root	Shoot	Root	Shoot	Root	Shoot
CN	0.76 ± 0.36d	3.02 ± 0.01d	0.04 ± 0.31h	0.23 ± 0.02i	24.4 ± 0.63f	32.7 ± 1.55e
BN	1.64 ± 0.28d	5.16 ± 0.02c	0.07 ± 0.25b	0.43 ± 0.02c	43.5 ± 1.41bc	40.5 ± 2.19c
FOP	0.87 ± 0.24d	5.39 ± 0.42c	0.06 ± 0.24d	0.33 ± 0.41f	32.7 ± 1.06d	40.5 ± 2.05c
CFOP	3.34 ± 0.06a	8.12 ± 0.02a	0.08 ± 0.09a	0.55 ± 0.03a	46.5 ± 1.41a	49.5 ± 0.7a
BT	1.85 ± 0.22bc	6.47 ± 0.28b	0.06 ± 0.24 c	0.43 ± 0.27c	41.5 ± 0.14c	45.0 ± 1.41b
BN + FOP	1.75 ± 0.22c	5.03 ± 0.49c	0.06 ± 0.19e	0.24 ± 0.48h	27.2 ± 0.42e	34.4 ± 1.90de
BN + CFOP	1.01 ± 0.05d	3.24 ± 0.41d	0.04 ± 0.08h	0.38 ± 0.41d	31.2 ± 0.49d	40.0 ± 1.48c
BN + BT	0.77 ± 0.09d	3.09 ± 0.44d	0.05 ± 0.13g	0.34 ± 0.41e	32.7 ± 1.69d	36.5 ± 0.70d
FOP + BT	2.33 ± 0.16b	7.41 ± 0.26a	0.07 ± 0.13b	0.46 ± 0.25b	44.6 ± 0.56ab	48.5 ± 0.70a
CFOP + BT	1.07 ± 0.16d	5.36 ± 0.28c	0.05 ± 0.16f	0.27 ± 0.24g	44.2 ± 1.41ab	40.5 ± 1.41c

Values are the average of 2 replicates ± SE. Different letters shows that the values are significantly different from each other.

**TABLE 5:** Proteins, Phenolics, and chlorophyll a, b concentrations in brinjal plant *(Solanum melongena)* as affected by various treatments: Control (CN); bentonite (BN); Iron Oxide nanoparticles (FOP); Cerium-doped iron oxide nanoparticles (CFOP); *Azotobacter nigricans* sp. (BT); BN + FOP; BN + CFOP; BN + BT; FOP + BT; T10 = CFOP + BT.

Treatments	Protein content (μ g g^–1^)			Phenolics content (μ g g^–1^)			Chlorophyll a (mg mL^–1^)	Chlorophyll b (mg mL^–1^)
	Root	Stem	Leaf	Root	Stem	Leaf	Leaf	Leaf
CN	8.91 ± 0.02i	8.50 ± 0.10h	6.55 ± 0.12h	51.6 ± 0.14e	52.2 ± 1.20f	53.2 ± 0.21d	1.20 ± 0.11e	2.15 ± 0.09f
BN	11.1 ± 0.17g	11.7 ± 0.07c	8.59 ± 0.22g	52.3 ± 1.13de	56.4 ± 1.83d	55.8 ± 0.91cd	4.88 ± 0.25b	4.71 ± 0.14bc
FOP	10.7 ± 0.15h	9.02 ± 0.07f	10.2 ± 0.20e	53.8 ± 0.49cd	53.3 ± 0.77ef	58.5 ± 4.87c	3.85 ± 0.07c	4.29 ± 0.29cd
CFOP	19.8 ± 0.05a	13.2 ± 0.07b	14.4 ± 0.15a	66.5 ± 0.14a	91.5 ± 0.42a	63.6 ± 0.14b	6.22 ± 0.05a	7.73 ± 0.41a
BT	9.05 ± 0.12i	9.59 ± 0.02e	8.84 ± 0.07fg	54.8 ± 0.91c	53.9 ± 0.35ef	53.4 ± 0.28d	2.29 ± 0.20d	3.25 ± 0.02e
BN + FOP	12.0 ± 0.05f	8.87 ± 0.05g	11.5 ± 0.05c	53.9 ± 1.06cd	52.7 ± 0.07ef	56.1 ± 0.14cd	4.04 ± 0.49b	4.16 ± 0.33d
BN + CFOP	12.4 ± 0.02e	8.05 ± 0.02i	10.8 ± 0.17d	54.1 ± 0.14c	59.5 ± 0.21c	58.2 ± 0.91c	4.53 ± 0.11e	3.23 ± 0.11e
BN + BT	14.9 ± 0.07c	8.73 ± 0.07g	9.05 ± 0.07f	54.1 ± 1.55c	54.2 ± 0.14e	55.2 ± 0.28c	4.53 ± 0.34b	4.71 ± 0.40bc
FOP + BT	17.4 ± 0.02b	17.2 ± 0.05a	12.0 ± 0.16b	61.2 ± 0.26b	65.4 ± 1.41b	72.6 ± 0.07a	5.72 ± 0.22a	5.14 ± 0.16b
CFOP + BT	14.5 ± 0.05d	11.1 ± 0.15d	11.2 ± 0.12cd	53.7 ± 0.63cd	83.0 ± 0.35ef	56.1 ± 0.14cd	2.19 ± 0.17d	2.81 ± 0.20e

Values are average of 2 replicates ± SE. Different letters shows that the values are significantly different from each other.

### Scenario of Pb and Cr concentrations in soil and plant

**FIGURE 3:**
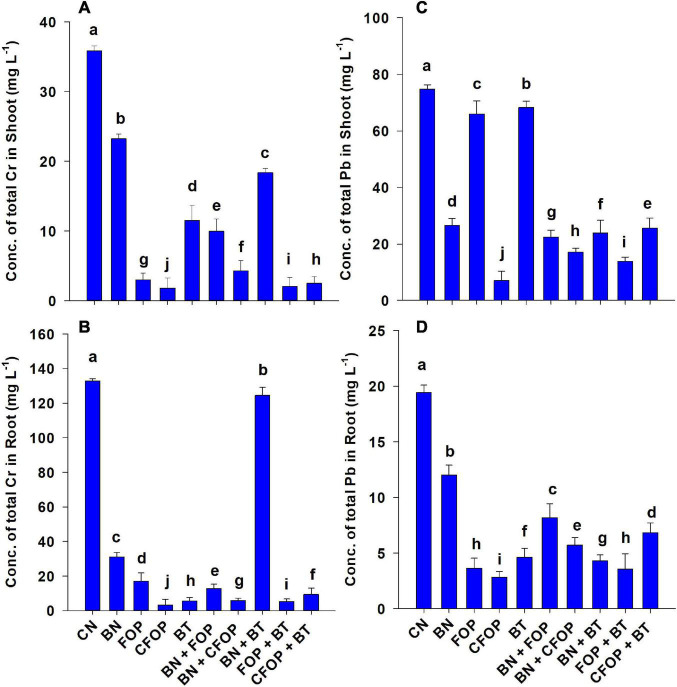
Concentrations of total Cr and Pb (mg L^−1^) in stem **(A,C)** and root **(B,D)** of brinjal plant (*Solanum melongena*) as affected by various treatments: Control (CN); bentonite (BN); Iron Oxide nanoparticles (FOP); Cerium-doped iron oxide nanoparticles (CFOP); Azotobacter nigricans sp. (BT); BN + FOP; BN + CFOP; BN + BT; FOP + BT; T10 = CFOP + BT. At (*P* < 0.05) column with changed letters are significantly different.

Wastewater application significantly (*P <* 0.01) induced Cr and Pb stress in soil and their accumulation in *Solanum melongena* L. (Figure 3). However, it was shown that the Cr and Pb stress was significantly (*P* < 0.01) tolerated through BN, BT, FOP, and CFOP applications (Figure 3). Bentonite played a vital role in combating tannery wastewater stress in *Solanum melongena* L. when applied alone and with bacteria. However, the concentration of Pb significantly (*P* < 0.01) decreased (90%) with the application of CFOP as compared with CN in the shoot of *Solanum melongena* L. Moreover, the concentration of shoot Cr competently decreased (94%) by the application of FOP + BT when compared with CN; however, *Azotobacter nigricans* sp. did not demonstrate efficient results when applied alone. However, the maximum decrease (99%) in Cr was observed in the root of the *Solanum melongena* L. through the foliar application of CFOP when compared with CN.

### Scenario of oxidative damage and antioxidants defense system

**FIGURE 4:**
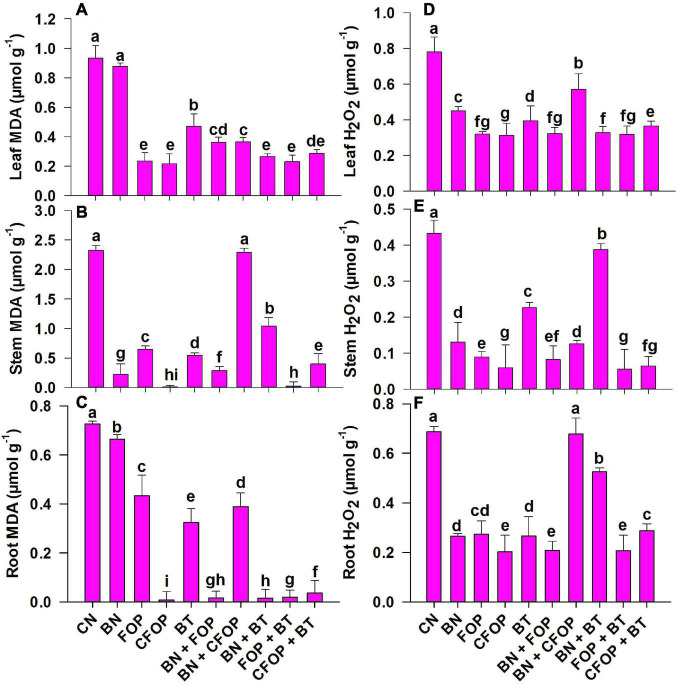
Malondialdehyde (MDA) and hydrogen peroxide (H_2_O_2_) concentrations in leaves **(A,D)**, stem **(B,E)** and root **(C,F)** of brinjal plant (*Solanum melongena*) as affected by various treatments: Control (CN); bentonite (BN); Iron oxide nanoparticles (FOP); Cerium-doped iron oxide nanoparticles (CFOP); Azotobacter nigricans sp. (BT); BN + FOP; BN + CFOP; BN + BT; FOP + BT; T10 = CFOP + BT. At (*P* < 0.05) column with changed letters are significantly different.

**FIGURE 5:**
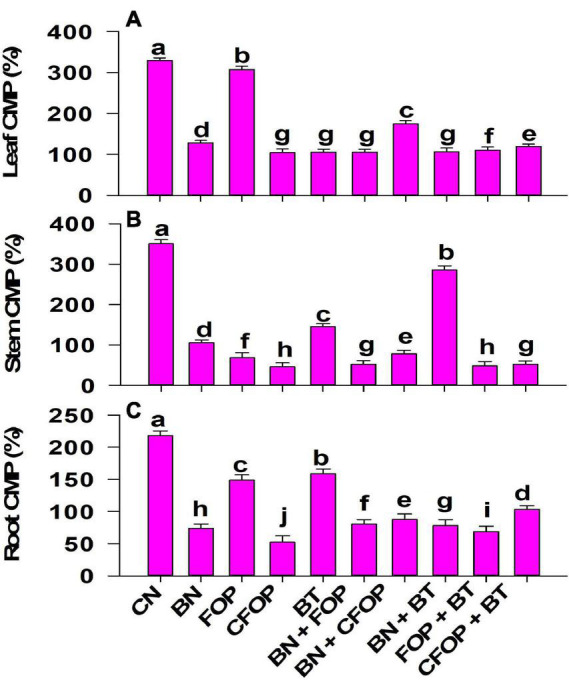
Cell membrane permeability [CMP (%)] in leaves **(A)**, stem **(B)**, and root **(C)** of brinjal plant (*Solanum melongena*) as affected by various treatments: Control (CN); bentonite (BN); Iron Oxide nanoparticles (FOP); Cerium-doped iron oxide nanoparticles (CFOP); Azotobacter nigricans sp. (BT); BN + FOP; BN + CFOP; BN + BT; FOP + BT; T10 = CFOP + BT. At (*P* < 0.05) column with changed letters are significantly different.

The application of tannery wastewater significantly (*P <* 0.01) induced oxidative stress through uncontrolled production of MDA, H_2_O_2_, and CMP in the *Solanum melongena* L. (Figures 4, 5). The addition of different treatments significantly (*P <* 0.01) influenced the enzymatic defense mechanism of *Solanum melongena* L., which effectively controlled oxidative damage in plant. However, the results were well pronounced with the foliar application of FOP and CFOP under tannery wastewater stress (Figures 4, 5); however, the application of CFOP revealed maximum reduction (95%) in the production of MDA as compared with CN in the stem of *Solanum melongena* L. (Figure 4). Interestingly, the application of BN + FOP significantly (*P <* 0.01) decreased (71%) the production of H_2_O_2_ in roots; maximum reduction (89%) in stem H_2_O_2_ was observed through CFOP as compared with CN under Cr and Pb stress (Figure 4). Moreover, a similar trend was investigated in CMP, where maximum reduction (85%) in stem cell membrane permeability was noticed under CFOP (Figure 5).

**FIGURE 6:**
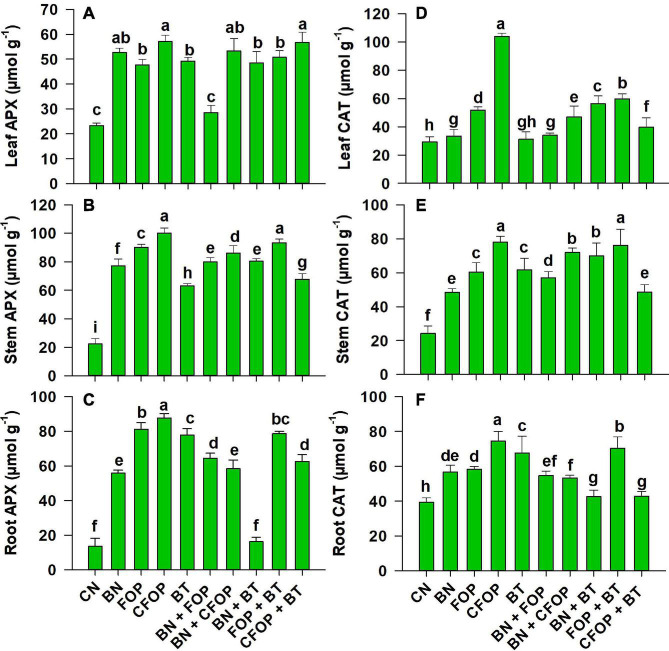
Ascorbate peroxidase (APX) and catalase (CAT) concentrations in leaves **(A,D)**, stem **(B,E)**, and roots **(C,F)** of brinjal plant (Solanum melongena) as affected by various treatments: Control (CN); bentonite (BN); Iron Oxide nanoparticles (FOP); Cerium-doped iron oxide nanoparticles (CFOP); Azotobacter nigricans sp. (BT); BN + FOP; BN + CFOP; BN + BT; FOP + BT; T10 = CFOP + BT. At (*P* < 0.05) column with changed letters are significantly different.

It was shown that the activities of ROS scavenging enzymes (APX and CAT) were significantly (*P <* 0.01) triggered by the application of different soil amendments and efficiently defended all parts of *Solanum melongena* L. under oxidative stress (Figure 6). However, wastewater application significantly caused oxidative damage by suppressing the activities of APX and CAT (Figure 6). So, activities of leaf APX are efficiently triggered (onefold) by the addition of CFOP and CFOP + BT relative to CN, while a competent increase (onefold) was observed in stem APX under the addition of CFOP relative to CN. Similarly, catalase activities were also significantly (*P <* 0.01) triggered in all parts of the *Solanum melongena* L. through the application of iron-oxide nanoparticles when applied alone and also after enrichment with cerium (Figure 6). Interestingly, the antioxidant defense mechanism was efficiently triggered by the significant (*P <* 0.01) increase (twofold) in stem catalase activity through the application of CFOP and FOP + BT when compared with CN (Figure 6). However, the first-order reaction was highly observed through scavenging burst production of reactive oxygen species (Figures 4–6).

### Scenario of secondary metabolites to regulate oxidative damage

Wastewater application significantly (*P <* 0.01) reduced the activities of non-enzymatic defense mechanisms, such as total phenolics and proteins (BSA), in all parts of the *Solanum melongena* L. (Table 5). However, the addition of BN, FOP, CFOP, and BT as heavy metal adsorbents effectively activated the phenolics and proteins to combat oxidative damage in the *Solanum melongena* L. under Cr and Pb stress (Table 5). Protein content was significantly (*P <* 0.01) increased (onefold) through the application of CFOP in the leaf of *Solanum melongena* L. relative to CN; a maximum increase (onefold) in stem protein was observed through the addition of FOP + BT when compared with CN (Table 5). Moreover, phenolic compounds significantly (*P <* 0.01) scavenged reactive oxygen species in *Solanum melongena* L. This might occur through more production of hydroxyl groups by applying different amendments. Interestingly, total phenolic compounds were also significantly (*P* < 0.01) increased (75%) through the application of CFOP in the stem of *Solanum melongena* L. relative to CN, but results were well pronounced with the application of FOP + BT in the leaf, which increased 35% phenolic content when compared with CN (Table 5).

## Discussion

### Scenario of different amendments to tolerate stress in soil and *Solanum melongena* L.

The effect of tannery wastewater was efficiently diminished by applying bentonite in soil (Table 2). This study demonstrated that increase in the concentration of bentonite effectively increased adsorption efficiency (38.54 mg g^–1^) of Cr removal (Table 2). This might be due to the higher number of available active/exchangeable sites through the addition of the highest rate of bentonite (6%) (Table 2). A similar trend was observed in which Cu and Pb ions were adsorbed onto the bentonite; this might be due to the accessibility of a higher surface area with a greater number of ion exchange locations when the initial concentration is constant (Tian et al., 2014; Hussain et al., 2021). Moreover, bentonite can immobilize PTEs in soil owing to its isomorphic substitution, negative charge, and environmental compatibility (Xie et al., 2018). The adsorption process of Cr was investigated by adding bentonite with 30 min interval (Table 2). Other factors, i.e., initial metal concentration, temperature, adsorbent mass, and pH, were constant throughout the experiment. So, it was revealed that bentonite’s percentage removal efficiency increased by increasing the contact time until equilibrium was attained at 30 h (Table 2). However, in the initial stages, the removal efficiency might be higher due to a larger number of available exchangeable sites (Table 2). Similarly, Hussain et al. (2021) observed that the process of ion exchange (Cu and Pb) is fast at the initial stage due to the presence of a larger number of active sites, which efficiently removed Cu (87%) and Pb (89%) through the application of bentonite relative to control; with time, the reaction reached the equilibrium stage (Bharwana et al., 2014; Khan et al., 2014; Daud et al., 2015).

Moreover, the adsorption process of bentonite was observed by applying Langmuir and Freundlich isotherm models to experimental data (Table 3). Comparison of the correlation factor (*R*^2^) of both these models depicted that the Langmuir adsorption isotherm model fitted best for this study (Table 3). However, a similar adsorption isotherm trend was observed in the adsorption of Cd ions on a monolayer of adsorbent (iron-doped biochar) (Dad et al., 2021). The application of tannery wastewater significantly (*P <* 0.01) induced Cr and Pb stress in *Solanum melongena* L. (Figure 3). The literature revealed that the compounds of Cr cause toxicity in plants, with negative effects such as wilting, necrosis, chlorosis, and ultimately death of affected plants; similarly, absorption of Pb in plants affects physiological and biochemical functions of plants, negatively affecting water balance and mineral content (Rehman et al., 2021). Moreover, the stress of heavy metals (Cr, Ni, Cd, Mn, As, Pb, Cu, and Hg) induce oxidative stress through uncontrolled production of ROS, which ultimately decreases chlorophyll content and overall growth of plants by suppressing the activities of enzymes (SOD, APX, and CAT), proteins, and phenolic compounds (Rehman et al., 2021). Similarly, Kumar and Pathak (2018) observed that Cu, Pb, Hg, Cr, and As cause structural and physiological changes in plants. However, the applications of CFOP, FOP + BT, and BN significantly (*P <* 0.01) regulated Cr and Pb ions in the *Solanum melongena* L. (Figure 3). The findings of this study were highly consistent with a similar study that sprayed iron nanoparticles (730 and 830 mgL^–1^) on the *Solanum villosum* plant, thus improving leaf area, antioxidant enzymes, chlorophyll, and protein contents in the plant (Ghaseminezhad et al., 2021). This reduction in metal concentration by CFOP and FOP may be attributed to the Phyto-stabilization of metals. Moreover, the reduction in the bioavailability of heavy metal ions by BN may be attributed to larger active sites of the adsorbent (bentonite) and its high adsorption capacity. Likewise, a recent study revealed that the concentrations of Cd (10 mgL^–1^) and Pb (20 mgL^–1^) were significantly reduced (49 and 52%, respectively) by the presence of bacterial strains (*Enterobacter bugandensis* XY1 and *Serratia marcescens* X43) through metal ions’ chelation in water spinach (Wang et al., 2022).

### Scenario of different amendments to stimulate growth parameters in *Solanum melongena* L.

Growth and physiology of *Solanum melongena* L. was significantly (*p <* 0.01) retarded under the application of tannery wastewater (Table 4). However, the fresh weight, length, and total biomass of *Solanum melongena* L. was effectively stimulated through BN, BT, FOP, and CFOP applications relative to CN (Table 4). Moreover, Cr and Pb ions in plants cause osmotic stress by binding their cell walls, thus reducing plants’ growth and development (Mauro et al., 2020). In this study, the application of Iron oxide nanoparticles (zero-valent and cerium-doped) significantly (*p <* 0.01) increased the biomass of the plant as compared to other amendments (Table 4). A similar study revealed that iron-oxide nanoparticles (0, 50, and 500 ppm) significantly improved the growth and physiology of the sunflower plant by binding toxic elements present in the soil during a 7-day experiment (Kornarzyński et al., 2020). Similarly, chlorophyll a and b significantly (*p* < 0.01) decreased under the application of tannery wastewater (Table 5). The addition of BN, BT, FOP, and CFOP significantly (*p <* 0.01) improved the chlorophyll content by tolerating Cr and Pb stress (Table 5). According to Morales et al. (2018), PTEs alter the movement of the electron transport chain, disturbing the normality of the thylakoid membrane and chloroplast, further minimizing the chlorophyll content in plants; similar findings were observed in this experiment (Table 5). However, according to literature, *Azotobacter* sp. acts as a bio-stimulant and bio-fertilizer, which stimulates the plant’s growth by providing some metabolites; moreover, it synthesizes indole acetic acid, phytohormones, gibberellin, and cytokinin which further improve the photosynthetic machinery of plant, hence causing improved growth and yield (Hindersah et al., 2020). Likewise, Rossi et al. (2017) investigated the efficiency of cerium oxide nanoparticles (0 and 500 mgKg^–1^) through enhanced activity of photosystem II in soybean and also improvement in its biomass and physiological parameters through tolerating Cd (0, 0.25, and 1 mg Kg^–1^) stress.

### Scenario of different amendments to combat oxidative damage through triggering enzymatic (APX and CAT) defense mechanism in *Solanum melongena* L.

Biotic and environmental factors trigger oxidative stress in plants, causing cell damage and dysfunction (Nawkar et al., 2013). It was investigated that the severe oxidative damage in *Solanum melongena* L. was caused by tannery wastewater irrigation. However, oxidative stress is mainly caused by disturbance in plant physiology to balance ROS generation and detoxification and overproduction of ROS to signal immunity response for defense and adaptation (Nawkar et al., 2013). A similar trend for bentonite has also been discussed in Tanzeem-ul-Haq et al. (2020), showing a high concentration of MDA reflecting severe PTEs stress conditions. Interestingly, the application of FOP in combination with BT and CFOP showed excellent performance through scavenging the burst production of ROS in the *Solanum melongena* L. (Figures 4, 5). This can be related to the high accumulation of water and nutrients under iron oxide nanoparticles, which increases plant physiological performance (Rizwan et al., 2019). Similar results were depicted with iron oxide nanoparticles (NPs) to reduce MDA and H_2_O_2_ concentration in root and shoot of wheat grown under PTEs stress. The literature revealed that the application of the Ce-NPs is beneficial in maintaining the cell structure as a low concentration of Ce might act as a catalyst in scavenging ROS, which ultimately preserves the chloroplast structure and cell wall (Rizwan et al., 2019; Jurkow et al., 2020).

In this study, APX and CAT were significantly (*p <* 0.01) influenced by the applications of BN, BT, FOP, and CFOP relative to control under the tannery’s wastewater stress (Figure 6). Among all antioxidant enzymes, CAT and APX are major enzymes for metabolizing ROS and controlling their harmful impacts on cell functioning (Anjum et al., 2016). Studies have shown that Cr-contaminated soil causes oxidative stress in plants, disrupting the oxidants and antioxidants balance of plants (Espina et al., 2014). APX and CAT are responsible for regulating the photosynthetic mechanism of the plant under adverse environmental conditions (Zaheer et al., 2019a,b; Gomes et al., 2022). Activities of APX and CAT enzymes in plants perform important H_2_O_2_ scavenging activities through more production of hydroxyl groups (Ahmad et al., 2020; Gomes et al., 2022). Results revealed that APX and CAT, both antioxidant enzymes, worked synergistically to activate plants’ defensive mechanism against ROS through FOP + BT and CFOP applications most competitively under Cr and Pb stress (Figure 6). Likewise, another study also observed significant activation of APX and CAT through the foliar applications of FOP in the *Dracocephalum moldavica* L. plant under 100 mM of NaCl stress (Moradbeygi et al., 2020).

### Scenario of different amendments to combat oxidative damage through triggering non-enzymatic (proteins and phenolics) defense mechanism in *Solanum melongena* L.

The content of protein and total phenolics significantly (*p <* 0.01) improved through the applications of BN, BT, FOP, and CFOP in the *Solanum melongena* L. under tannery wastewater stress (Table 5). Abou-Hassan et al. (2018) observed that the increased rate of proteins denaturation under PTEs stress led to reduced biochemical compounds such as lipids, carbohydrates, nucleic acid, and phenolics in maize plants. While, the addition of BN, BT, FOP, and CFOP significantly (*p <* 0.01) triggered non-enzymatic mechanisms by producing a greater number of hydroxyl ions (OH^–^) (Feng et al., 2018), which further regulated the effect of ROS in *Solanum melongena* L. (Table 5). However, results were well pronounced with CFOP and FOP + BT applications to tolerate Cr and Pb stress by stimulating proteins’ activation in the *Solanum melongena* L. (Table 5). Similarly, Moradbeygi et al. (2020) revealed that flavonoid, total phenolic, and anthocyanin contents were efficacious with the foliar application of iron oxide nanoparticles in *Dracocephalum moldavica* L. under NaCl stress (100 mM). So, it would be recommended to utilize FOP and CFOP in association with BT to regulate the toxic effects of tannery wastewater in developing countries, including Pakistan.

## Conclusion

The present study verified that tannery wastewater application significantly (*p <* 0.01) induced Cr and Pb stress in soil, which disturbed the overall growth and physiology of *Solanum melongena* L.; however, the applications of BN, BT, FOP, and CFOP effectively recovered the oxidative damage in *Solanum melongena* L., thus improving its growth and physiology. This might cause benefits in stress tolerance, improved cell structures, triggered defense systems, and improved crop yield. Hence, it was demonstrated that the application of CFOP and FOP + BT more competitively resolved the issue of tannery wastewater toxicity by improving soil fertility and crop yield in a sustainable way.

## Data availability statement

The original contributions presented in this study are included in the article/Supplementary material, further inquiries can be directed to the corresponding author/s.

## Author contributions

WK conceived the idea and designed the research. SG, UR, WK, and HA conducted the experiment. XW analyzed the data. MW, FZ, and BA remained involve in synthesis of FOP, CFOP nanoparticles X-ray measurements and evaluations of these nanoparticles. All authors contributed to the subsequent development, approved the final manuscript, and again reviewed the manuscript carefully.
