# Supplementary material for: Investigating the role of bentonite clay with different soil amendments to minimize the bioaccumulation of heavy metals in Solanum melongena L. under the irrigation of tannery wastewater
Source: Front Plant Sci. 2022 Sep 29;13:958978. doi: 10.3389/fpls.2022.958978 (PMC9558103; doi:10.3389/fpls.2022.958978)
Supplement: Supplementary file 1 [file Table_1.DOCX]

**Supplementary data**

| **Heavy Metals** | **10%**  **(mgL^-1^)** | **20%**  **(mgL^-1^)** | **30%**  **(mgL^-1^)** | **50%**  **(mgL^-1^)** | **75%**  **(mgL^-1^)** | **100%**  **(mgL^-1^)** |
| --- | --- | --- | --- | --- | --- | --- |
| **Cr** | 132 | 219 | 291 | 407 | 489 | 923 |
| **Pb** | 1.879 | 2.867 | 4.795 | 5.672 | 7.245 | 10.547 |
| **Cu** | 0.138 | 0.145 | 0.166 | 0.204 | 0.235 | 0.278 |
| **Ni** | 0.052 | 0.197 | 0.322 | 0.815 | 1.214 | 1.627 |
| **Mn** | 0.037 | 0.133 | 0.233 | 0.520 | 0.709 | 0.872 |
| **Cd** | 0.036 | 0.066 | 0.095 | 0.136 | 0.194 | 0.234 |

**Table S1.** Heavy metals concentration in Tannery's wastewater.
